# Oral Ethanol for Methanol Poisoning: A Case Report

**DOI:** 10.31729/jnma.v63i2091.9230

**Published:** 2025-11-30

**Authors:** Rony Maharjan, Kripa Maharjan

**Affiliations:** 1 Department of General Practice and Emergency Medicine, Patan Academy of Health Sciences

**Keywords:** *ethanol*, *methanol*, *poisoning*

## Abstract

Methanol poisoning usually occurs due to accidental ingestion of distilling and fermenting errors or beverage contamination. The typical presentation includes visual disturbances with severe metabolic acidosis and neurological damage.

A 32-year-old male presented with blurring of vision for 2 days, with a history of local alcohol consumption 2 days prior, with severe metabolic acidosis on arterial blood gas analysis, and was clinically diagnosed and treated as methanol poisoning in the absence of a serum methanol assay. He responded favorably to the treatment with timely oral ethanol via Nasogastric tube (56 grams, 0.8 g/kg body weight) followed by hemodialysis. His bedside visual acuity was normal, and his complaint of blurred vision subsided on the following day of treatment. This case demonstrates that timely empirical treatment based on clinical suspicion can be life-saving in methanol poisoning, even in the absence of typical diagnostic markers or access to standard antidotal therapy.

## INTRODUCTION

Alcohol is commonly abused substance globally, with its true burden underestimated due to underreporting. Outbreaks in several countries show case fatality rates of 8% to over 30%.^1,2,3^

In Nepal, alcohol includes homemade or illicit beverages, leading to accidental ingestion of toxic alcohols, mainly methanol.^4^ Though cases and deaths are reported in Nepal, national data are unavailable.^5,6^ Methanol toxicity results from formic acid formation and is treated with fomepizole or ethanol. ^7^ Study in Kathmandu valley reported of 20.7 % prevalence, with ethanol available at 4 non-teaching hospitals and fomepizole at only one.^8^

We report a case of methanol poisoning, highlighting management, clinical challenges, and decision-making strategies in low-resource settings.

## CASE REPORT

A 32-year-old male from Kathmandu valley presented to the Eye Outpatient Department with complaints of blurring of vision in both eyes and shortness of breath for 2 days. After evaluating the case, he was then referred to the medical outpatient department and eventually to the Emergency department. On eliciting the history further, the blurring of vision was diffuse and painless.

There was no history of fever, headache, vomiting, altered mental status, loss of consciousness or power, abdominal pain, trauma, vertigo, or dizziness. His bowel and bladder habits were normal. He gave a history of daily consumption of local alcohol and had consumed 2 days prior as well. He smoked 10 pack years. There was no significant past comorbidity.

On arrival at the emergency, his airway was clear, his respiratory rate was high but he was maintaining adequate saturation, and circulation was stable. His vitals were:- Pulse rate: 94 beats per minute, respiratory rate: 24 breaths per minute, temperature :97.5°F, SpO2: 97% in room air, and blood pressure: 110/80 mmHg. He was oriented to time, place and person, with a Glasgow Coma Scale of 15/15. No significant findings were observed on exposure. There was no pallor, icterus, cyanosis, clubbing, edema, dehydration, or lymphadenopathy. There was no odor of alcohol on examination. His initial findings of eye examination showed visual acuity of 6/60 in both eyes, with best corrected visual acuity (BCVA) of 6/6 pinhole and 6/9 in the right and left eye, respectively; and intraocular pressure of 17 mm Hg. Extraocular movements were full in all directions of gaze, free and painless; the pupil was bilaterally equal and reactive to light, and corneal reflexes were intact. His fundus evaluation under full dilation showed normal findings.

The rest of his systemic examinations were normal, including the Cardio-Respiratory and Central Nervous System (CNS) examination. Electrocardiogram showed sinus rhythm with a heart rate of 95 beats per minute, normal axis, and normal echocardiography. His Arterial Blood Gas (ABG) analysis showed severe metabolic acidosis, osmolar gap of less than 10 with values and progression as shown in the Table 1.

On the basis ofhistory, examination, and lab values, a provisional diagnosis of methanol poisoning was made in the absence of serum methanol level, and the patient was then managed with oral ethanol due to the unavailability of IV ethanol and fomepizole. Oral ethanol in the loading dose of 0.6-0.8 grams per kilogram of the patient’s body weight was given via Nasogastric tube.^9^

Patient’s weight was 70gm, so the loading dose of 56 gm of ethanol (120 ml of 40% Vodka). He also received an intravenous sodium bicarbonate 300 mEq bolus dose in an emergency for severe metabolic acidosis.

The patient was then admitted to the medical intensive care unit (MICU). His blood investigations were, which showed raised total leukocyte counts of 20,300 cells per μl, which gradually decreased on the following day of admission to 16,000 cells per μl. The rest of his blood investigations, including liver function test, renal function test, sodium, potassium, calcium, magnesium, phosphorus, HIV, HbsAg/HCV, and urine routine were normal. His ESR was 18, and C reactive protein was 2.3. His ABG showed severe metabolic acidosis with values and progression as shown in the Table 1.

After the patient shifted to the Medical Intensive Care Unit, he underwent hemodialysis after 4 hours of admission for severe metabolic acidosis. Urine drug screen was sent to rule out other poisoning for Tramadol, Amphetamine, Opiates, Cocaine, Barbiturates, Tetrahydrocannabinol (THC), Benzodiazepines, Methadone, and Morphine, all of which were not detected. Serum methanol level couldn’t be assessed due to the unavailability of the test at any centers during that time. MRI brain showed acute lacunar infarcts in bilateral posterior putamen with thickened, edematous bilateral optic nerve with blurring of margins, involving the intraorbital segment, likely optic neuritis.

On day 1 of admission, he had no complaints of blurred vision. Ophthalmology consultation was done and bedside visual acuity was normal in both eyes. Anterior segment was quiet and posterior segment showed normal findings. Extraocular movements were full range in all directions, fundus optic disc showed normal margin and color and cup to disc ratio: 0.3 bilaterally. The patient was advised to receive intravenous methylprednisolone 1-gram STAT dose with tapering dose in view of MRI findings, which was refused by the patient.

During his MICU stay, patient received folic acid 50 mg thrice daily as management for methanol toxicity, thiamine 100 mg once daily, and pantoprazole 40 mg once daily for harmful alcohol use.

On day 3 of admission, patient was shifted to the General medical ward, and the patient was discharged on request on day 5 of admission with thiamine and advised to follow-up in ophthalmology, medicine, and psychiatric outpatient department (OPD). However, the patient lost to follow-up in OPD as advised. On enquiring about his health condition after a week of hospital discharge over the phone, the patient said he had no complaints and couldn’t follow up due to his daily duties.

**Table 1:** Serial Arterial Blood Gas (ABG) Analysis from May 12 to May 14, 2025

	12/05/2025	13/05/2025	13/05/2025 (after 6 hours)	14/05/2025
ph	7.13	7.48	7.44	7.41
pCO2 mmHg	6.4	27.3	36.8	29.3
pO2 mmHg	124	109.3	118	87
HCO3 mmol/L	2.2	20.9	24.8	20.7
Lactate mmol/L	2.7	1.7	1.6	1.3
Base excess mmol/L	-23.2	-2.6	1.1	-4.7
Anion gap mEq/L	25.8	5.1	15.7	17.1
Serum Osmolality	276			

## DISCUSSION

Methanol poisoning is a life-threatening condition resulting from the ingestion of methanol, often found in adulterated alcoholic beverages or industrial products. The potentially lethal dose of methanol is 1 gram per kg, however even 30 ml ingestion can lead to potential visual damage.^9^ The toxicity arises from methanol’s metabolism to formic acid, which leads to severe metabolic acidosis, central nervous system depression, and multi-organ failure, leading to coma and death. The hallmark of methanol poisoning is visual impairment due to demyelination, and thus optic nerve atrophy, due to the direct toxic effect of byproduct formic acid which inhibits cytochrome oxidase, leading to decreased ATP production and impaired ATP-dependent functions. Inhibition of the sodium-potassium membrane pump disrupts electrical conduction, resulting in early but potentially reversible visual disturbances.^9^ The predominance of effect in optic nerve and CNS is assumed to be due to the high energy dependence of the functional profile of these tissues, which is responsible for the much greater sensitivity to mitochondrial toxins such as formic acid.^10^

The oxidation of methanol is generally three to six times slower compared to ethanol. Formic acid suppresses the cytochrome system, inhibiting the oxidative phosphorylation, thereby causing adenosine triphosphoric (ATP) acid deficiency that mainly occurs in the brain tissue and the retina. As the synthesis of ATP terminates, tissue hypoxia develops, leading to accumulation of lactic acid. This aggravates the systemic acidosis. In addition, methanol is strong protoplasmic poison that disrupts oxidative phosphorylation in the cytochrome oxidase system.^11^

Methanol ingestion should be considered in patients presenting with altered mental status, visual disturbance, gastrointestinal symptoms, dyspnea, chest pain following intake of alcohol. Laboratory values show unexplained anion gap metabolic acidosis.^12^ Increased osmolar gap suggests presence of osmotically active substance like alcohol, however it typically elevates early after ingestion and returns to normal, as metabolic acidosis develops ^13^ as seen in our patient. The presentation of blurred vision along with history of local alcohol ingestion raised suspicion in our case which was supported by the increased anion gap metabolic acidosis. Though the confirmation is by serum or urine methanol level, the test was not available in our context.

Patient was managed with oral ethanol due to unavailability of IV formulations and antidote fomepizole followed by Hemodialysis. Intravenous Ethanol (10% Formulary) if available is given as Loading dose of 8 mL/kg over 30 to 60 minutes. Maintenance dose of 1 to 2 mL/kg per hour was given and doubled during dialysis. The dose for oral Ethanol (50% or 100 proof): Loading dose of 2 mL/kg and maintenance dose of 0.2 to 0.4 mL/kg per hour, doubled during dialysis with therapeutic Serum Concentration target of 80 to 120 mg/dL. Oral ethanol can cause gastric irritation ,aspiration which was mitigated in our patient via Nasogastric administration. Besides the antidote, hemodialysis enhance the methanol and formate elimination, along with correction of metabolic acidosis Indications for hemodialysis include new visual impairment with metabolic acidosis, methanol concentration >50 mg/dL, severe metabolic acidosis refractory to resuscitation, ingestion of a lethal dose (1 gm/kg), renal failure. ^13^

Studies have shown that folic acid supplementation can reduce the toxic effects of methanol and enhance formate oxidation. Formate oxidation depends on hepatic tetrahydrofolate levels, which are regulated by two key factors: the availability of dietary folic acid and the efficiency of tetrahydrofolate regeneration during formate oxidation. Intravenous folinic acid is administered at doses 1 mg/kg, every 4 hourly. If folinic acid is not available, folic acid at the same dosage can serve as an alternative. ^9^

Methanol poisoning is a critical public health issue in Nepal, driven by unregulated local alcohol production and low public awareness. There is an urgent need for strict regulation of alcohol manufacturing, increased community education, establishment of poison information centers, and improved access to antidotes like ethanol or fomepizole to reduce fatalities. Current limitations include the lack of methanol assay facilities, absence of long-term patient follow-up, and no routine serum ethanol monitoring, which hinder effective management. Future efforts should focus on developing Nepal-specific toxicology protocols and strengthening laboratory and clinical infrastructure to address these unique challenges .^5^

## CONCLUSION

In low-resource settings, where diagnostic tests and antidotes for methanol poisoning are unavailable, clinical management relies on recognizing key symptoms such as severe metabolic acidosis, visual impairment, and altered consciousness, often following ingestion of homemade or adulterated alcohol. Empirical treatment based on clinical suspicion and supportive care, including use of oral ethanol if IV ethanol formulation is not available, and hemodialysis when indicated, can improve outcomes. This case also highlights the need for increased awareness among public regarding methanol poisoning and strict legislations for illicit liquor production and availability. In addition, the current challenges of the unavailability of specific tests for methanol poisoning, antidote supply in Nepal, requires prompt action.
